# A Gammaherpesvirus Complement Regulatory Protein Promotes Initiation of Infection by Activation of Protein Kinase Akt/PKB

**DOI:** 10.1371/journal.pone.0011672

**Published:** 2010-07-21

**Authors:** Beatrix Steer, Barbara Adler, Stipan Jonjic, James P. Stewart, Heiko Adler

**Affiliations:** 1 The Institute of Molecular Immunology, Clinical Cooperation Group Hematopoietic Cell Transplantation, Helmholtz Zentrum München - German Research Center for Environmental Health, Munich, Germany; 2 Max von Pettenkofer-Institute, Ludwig-Maximilians-University Munich, Munich, Germany; 3 Department of Histology and Embryology, Faculty of Medicine, University of Rijeka, Rijeka, Croatia; 4 Centre for Comparative Infectious Diseases, Department of Medical Microbiology, University of Liverpool, Liverpool, United Kingdom; University of Hong Kong, Hong Kong

## Abstract

**Background:**

Viruses have evolved to evade the host's complement system. The open reading frames 4 (ORF4) of gammaherpesviruses encode homologs of regulators of complement activation (RCA) proteins, which inhibit complement activation at the level of C3 and C4 deposition. Besides complement regulation, these proteins are involved in heparan sulfate and glycosaminoglycan binding, and in case of MHV-68, also in viral DNA synthesis in macrophages.

**Methodology/Principal Findings:**

Here, we made use of MHV-68 to study the role of ORF4 during infection of fibroblasts. While attachment and penetration of virions lacking the RCA protein were not affected, we observed a delayed delivery of the viral genome to the nucleus of infected cells. Analysis of the phosphorylation status of a variety of kinases revealed a significant reduction in phosphorylation of the protein kinase Akt in cells infected with ORF4 mutant virus, when compared to cells infected with wt virus. Consistent with a role of Akt activation in initial stages of infection, inhibition of Akt signaling in wt virus infected cells resulted in a phenotype resembling the phenotype of the ORF4 mutant virus, and activation of Akt by addition of insulin partially reversed the phenotype of the ORF4 mutant virus. Importantly, the homologous ORF4 of KSHV was able to rescue the phenotype of the MHV-68 ORF4 mutant, indicating that ORF4 is functionally conserved and that ORF4 of KSHV might have a similar function in infection initiation.

**Conclusions/Significance:**

In summary, our studies demonstrate that ORF4 contributes to efficient infection by activation of the protein kinase Akt and thus reveal a novel function of a gammaherpesvirus RCA protein.

## Introduction

Viruses use a variety of strategies to evade the host's immune response [1], [2]. A host mechanism involved in innate immunity against viruses is the complement system. Consequently, viruses have evolved to evade the actions of the complement system, thereby avoiding destruction by complement-mediated mechanisms [3]–[6]. A number of viruses not only avoid inactivation and destruction by complement but also use complement receptors to initiate infection. For example, EBV infects its target cell, the B cell, via complement receptor type 2 (CR2) [7].

The poxviral complement control protein VCP (vaccinia virus complement control protein) can bind to complement components C3b and C4b, respectively, thereby inactivating complement components or blocking the formation of the C3 convertase complex [8]. Extracellular vaccinia virus is resistant to complement because of incorporation of host complement control proteins into its envelope [9]. Herpesviruses encode complement regulatory proteins that can block complement activation and neutralization of virus particles [3]. For example, HSV-1 glycoprotein gC prevents complement-mediated cell lysis and virus neutralization [10], [11]. The open reading frame 4 (ORF4) of gammaherpesviruses, including human herpesvirus 8 (HHV-8; KSHV), herpesvirus saimiri (HVS), murine gammaherpesvirus 68 (MHV-68) and rhesus rhadinovirus (RRV), encode homologs of host regulators of complement activation (RCA) proteins [12]–[17]. MHV-68, HVS and RRV RCA proteins inhibit complement activation at the level of C3 and C4 deposition [15], [18]–[21]. The KSHV complement control protein (KCP) accelerates the decay of classical C3 convertase and induces the degradation of activated complement factors C4b and C3b [22]. The MHV-68 RCA protein has been shown to play an important role in viral evasion of complement in acute, persistent and latent infection in vivo [23].

Besides complement regulation, viral RCA proteins may have additional functions. For example, the poxvirus B5R protein is essential for virion morphogenesis and is also involved in polymerization of actin on virions in infected cells [24]. HSV gC may play a role in infection by interacting with heparan sulfate or attaching to polarized epithelial cells [25], [26]. Similarly, the RCA proteins of the gammaherpesviruses KSHV and MHV-68 have also been shown to interact with heparan sulfate and glycosaminoglycans [27]–[29]. In addition, the MHV-68 RCA protein has very recently been shown to facilitate MHV-68 replication in primary macrophages in a complement independent manner [30]. Studies to investigate the function of KSHV ORF4 during lytic infection are limited by the lack of a cell culture system capable of supporting productive replication. In contrast, MHV-68 replicates in conventional tissue culture systems and thus provides a model to study de novo gammaherpesvirus infection. MHV-68 is a natural pathogen of wild rodents [31]. The nucleotide sequence of MHV-68 is very closely related to KSHV [14]. Therefore, the functional roles of conserved gammaherpesvirus proteins can be addressed by mutagenesis of the corresponding viral genes [32].

Here, we made use of MHV-68 to study the role of ORF4 during infection of fibroblasts. While attachment and penetration of virions lacking the RCA protein were not affected, we observed a delayed delivery of the viral genome to the nucleus of infected cells, consistent with our previously published findings (30) that deletion of ORF4 results in delayed replication of MHV-68 in fibroblasts. Analysis of the phosphorylation status of a variety of kinases in infected cells revealed a significant reduction in the phosphorylation of the protein kinase Akt in cells infected with ORF4 mutant virus, when compared to cells infected with wt virus. Consistent with a role of Akt activation in the initial stage of the infection, inhibition of Akt signaling by the specific inhibitor Triciribine in cells infected with wt virus resulted in a phenotype closely resembling the phenotype of the ORF4 mutant virus. Pre-activation of Akt by addition of insulin partially reversed the phenotype of the ORF4 mutant virus. Importantly, the homologous ORF4 of KSHV was able to rescue the phenotype of the MHV-68 ORF4 mutant, indicating that ORF4 is functionally conserved and that ORF4 of KSHV might have a similar function in infection initiation.

## Results

### Delayed infection kinetics of ORF4 mutants as determined by FACS analysis and quantitative RT-PCR

**Figure 1:**
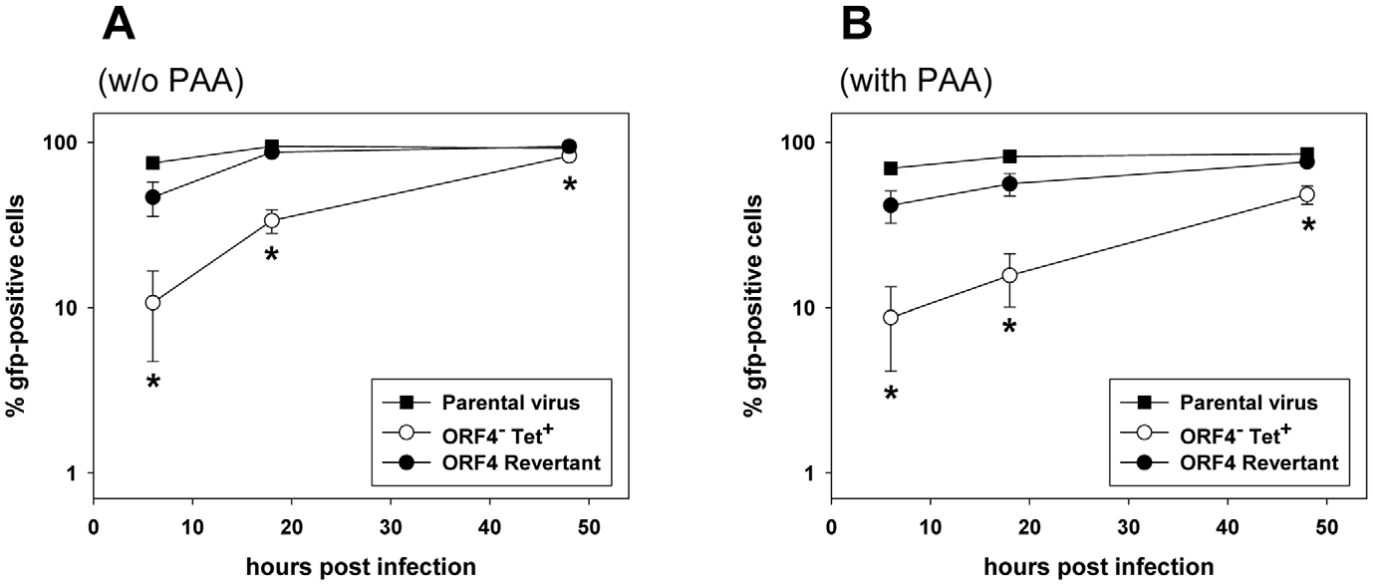
Kinetics of gfp-expression after infection of NIH3T3 cells with parental virus, the deletion mutant ORF4^−^Tet^+^ or the ORF4 revertant. Cells were infected at a multiplicity of infection of 10 for 1h at 37°C. Then, the inoculum was removed and fresh medium, without (panel A) or with (panel B) phosphonoacetic acid (PAA), was added. Cells were harvested immediately thereafter and after 6, 18 and 48 hours, respectively, and analyzed for gfp expression by FACS analysis. Data shown are means ± SD of three independent experiments. The asterisks indicate that the expression of gfp is significantly delayed after infection with the ORF4^−^Tet^+^ mutant, when compared to parental or revertant virus (p<0,02; Two-way ANOVA).

We had previously reported that the ORF4^−^Tet^+^ mutant showed delayed growth in fibroblasts in cell culture when compared to the parental virus [32]. Importantly, the corresponding ORF4 revertant displayed wt growth properties, indicating that the delayed growth of the ORF4^−^Tet^+^ mutant was due to deletion of ORF4 and not due to rearrangements outside ORF4 [32]. Now, we report on experiments aimed to discover the underlying mechanism(s). We hypothesized that one reason for the delayed growth might be that mutation of ORF4 affects initial stages of infection. To investigate this in more detail, we made use of expression of the green fluorescent protein (gfp), an inherent property of the recombinant viruses used [32]. The gfp is driven by the HCMV ie promoter and thus, its expression is independent of the replication of the viral genome and starts once the viral genome has reached the nucleus. NIH3T3 cells were infected with parental virus, ORF4^−^Tet^+^ mutant and ORF4 revertant at a multiplicity of infection of 10 for 1h at 37°C. Then, the inoculum was removed and fresh medium, with or without PAA, which inhibits DNA synthesis, was added. Cells were harvested immediately thereafter or after 6, 18 and 48 hours, and analyzed for gfp expression by FACS analysis. As expected, cells harvested immediately after infection were negative for gfp, indicating that gfp was not directly delivered to the infected cells by input virus but that gfp expression starts only after the viral genome has reached the nucleus (data not shown). After infection with the ORF4^−^Tet^+^ mutant, the increase in the number of gfp-positive cells was significantly delayed, when compared to the infection with parental virus or ORF4 revertant (Fig. 1A). The reduction was most pronounced at the early time points, consistent with the growth kinetics described previously [32]. To further support our hypothesis that mutation of ORF4 might affect an initial stage of infection and thus is independent from virus replication, we performed the same experiments in the presence of PAA. As shown in Fig. 1B, the results were very similar to those obtained in the absence of PAA. The reduced number of gfp-positive cells after infection with the ORF4^−^Tet^+^ mutant was not specific for NIH3T3 cells since it was also observed in mouse embryonic fibroblasts (MEF) (Fig. S1). Furthermore, it was not due to the presence of the tetracycline resistance gene in the mutant ORF4^−^Tet^+^, because mutant ORF4^−^Tet^−^, in which the tetracycline resistance gene has been removed, showed the same phenotype (Fig. S1).

**Figure 2:**
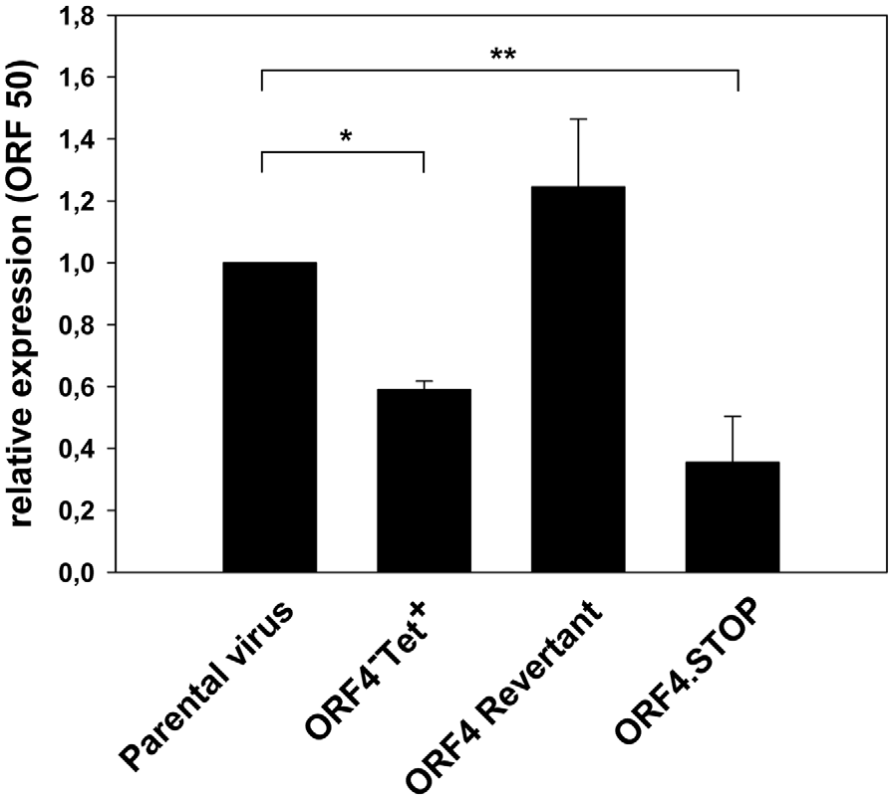
Delayed infection kinetics of the ORF4^−^Tet^+^ and ORF4.STOP mutants as determined by quantitative RT-PCR. The expression of the mRNA of the MHV-68 immediate-early gene Rta (ORF50) was examined by quantitative RT-PCR. Cells were infected at a multiplicity of infection of 10 for 1h at 37°C. Then, the inoculum was removed and fresh medium with PAA was added. Total RNA was isolated from infected cells 18 hours after infection and reverse transcribed. The resulting cDNA was used as template for PCR amplification of L8 and MHV-68 ORF50, respectively. Data are presented as relative expression of ORF50 after normalization to the corresponding L8 levels using the delta-delta C_t_ method. 18 hours after infection, the expression of the ORF50 mRNA was significantly reduced (*, p = 0,002; **, p = 0,025; Student's t-test) in cells infected with the ORF4 mutants when compared with the parental or revertant virus. Data shown are means ± SD of two independent experiments, each determined in duplicates.

To exclude the possibility that protein translation and thus gfp-expression is not equivalent between ORF4^−^Tet^+^ mutant and parental or ORF4 revertant virus, we investigated the infection kinetics of the ORF4 mutant at the mRNA level by quantitative real-time RT-PCR. For this purpose, we examined the expression of the message of the MHV-68 immediate-early gene Rta (ORF50) [33] which can only be transcribed after the viral genome has reached the nucleus. 18 hours after infection, the amount of detectable ORF50 mRNA was significantly lower in cells infected with the ORF4^−^Tet^+^ mutant, when compared to the parental or to the corresponding revertant virus (Fig. 2). In addition, an independently constructed ORF4.STOP virus showed a similar phenotype. Taken together, the RT-PCR data are consistent with the results from the FACS analysis, and both are indicative for a delayed delivery of the virus genome to the nucleus of the infected cell. We concluded from these data that the similarity of the phenotype of two ORF4 mutant viruses constructed independently is strongly suggestive for the phenotype being due to mutation of ORF4.

To further support this hypothesis and to exclude the possibility that mutation of ORF4 affected the transcription of neighbouring genes, we analyzed their transcription by quantitative real-time RT-PCR. As shown in Fig. S2A, mutations introduced into ORF4 did not change transcript levels from the adjacent genes M4 and ORF6.

### Attachment and penetration of ORF4 mutants

The experiments thus far supported our hypothesis that the mutation of ORF4 most likely affects an initial step of the infection process and not a late step which occurs after the viral genome has reached the nucleus and which requires DNA replication. Initial steps of herpesvirus infection include attachment, penetration, uncoating, transport of the capsids to and release of the viral genome into the nucleus [34]. Therefore, we next performed experiments analyzing attachment and penetration by different methods as described in experimental procedures. For both processes, we could not detect appreciable differences between ORF4^−^Tet^+^, parental virus and ORF4 revertant (Table S1 and Fig. S3).

### Delayed infection kinetics of the ORF4^−^Tet^+^ and ORF4.STOP mutants as determined by fluorescence microscopy

**Figure 3:**
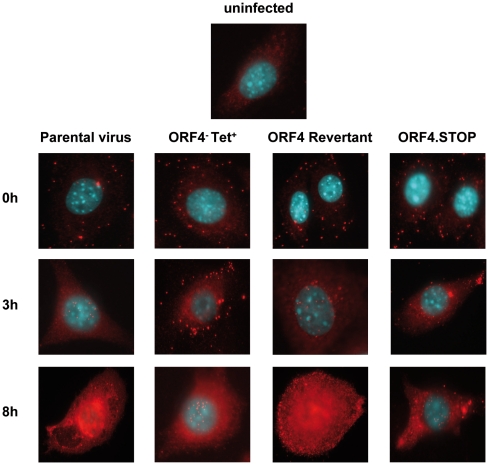
Delayed infection kinetics of the ORF4^−^Tet^+^ and ORF4.STOP mutants as determined by fluorescence microscopy. For immunofluorescence staining, NIH3T3 cells were seeded on coverslips in 12 well plates the day before staining. Cells were infected at a multiplicity of infection of 5–10 for 1h at 4°C to allow adsorption. Then, cells were washed three times and were either immediately (0h – upper panel) fixed with 4% paraformaldehyde or prewarmed medium was added and the cells were further incubated at 37°C. Three hours later (3h – median panel) or eight hours later (8h – lower panel), cells were fixed and permeabilized with 0.1% Triton-X100. Virus particles were visualized with polyclonal rabbit anti-MHV-68 antiserum followed by goat anti-rabbitCy3 antibody. Stained cells were mounted in ProLong® Gold antifade reagent with DAPI and analysed by using a Zeiss Axiovert 200M microscope. Cells were viewed with a 100× oil immersion objective. The top panel shows staining of uninfected cells as control. One representative experiment of 5 is shown.

Since attachment and penetration of ORF4 mutant viruses were not affected, we wanted to investigate the fate of the viruses in the subsequent phase of infection. For this purpose, we visualized virus particles immediately after adsorption and 3h post infection by staining with a polyclonal rabbit anti-MHV-68 antiserum and fluorescence microscopy. Immediately after adsorption, comparable numbers of equally distributed parental, revertant and ORF4 mutant virus particles were observed (Fig. 3, 0h). After 3h, however, parental and revertant virus particles accumulated around the nucleus of infected cells (Fig. 3, 3h). In contrast, both the ORF4^−^Tet^+^ and ORF4.STOP mutant virus particles appeared mainly localized in the periphery. At 8h post infection, newly synthesized viral proteins were already recognized by the anti-MHV-68 antiserum in cells infected with parental and revertant virus, respectively (Fig. 3, 8h). On the contrary, at this time, ORF4^−^Tet^+^ and ORF4.STOP mutant virus particles were now detectable around the nucleus, almost like parental and revertant viruses were at 3h post infection. In addition, de novo synthesis of viral proteins appeared just to be at the beginning, also indicative of a delayed infection kinetics. We concluded from these data that viruses lacking the RCA protein are less efficient in their ability to accumulate around the nucleus of infected cells.

### Determination of the phosphorylation status of key cellular kinases

**Figure 4:**
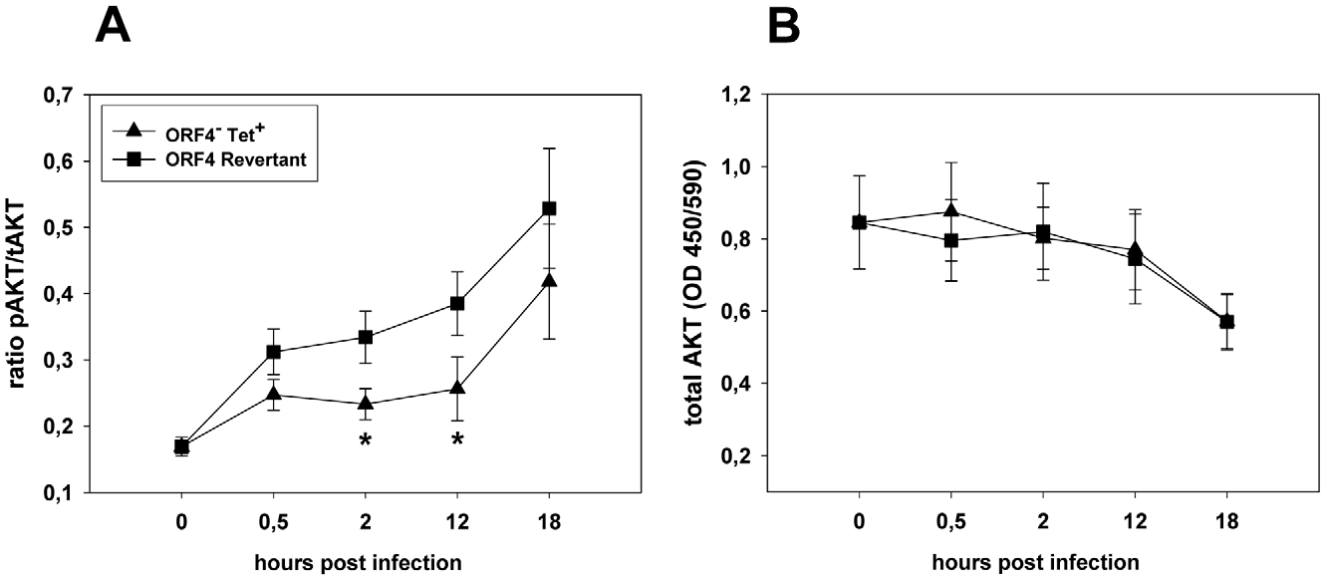
The relative phosphorylation level of the protein kinase Akt is significantly reduced after infection with the deletion mutant ORF4^−^Tet^+^. Cells were infected at a multiplicity of infection of 10 or left uninfected ( = time point 0). At the indicated time points after infection, the relative phosphorylation levels of the protein kinase Akt were determined by a cell-based protein phosphorylation ELISA. The data are presented as the amount of activated (phosphorylated) Akt protein (pAkt) relative to total Akt protein (tAkt) (panel A). Total Akt levels are additionally depicted in panel B. Data shown are means ± sem of three to seven independent determinations, each performed in triplicate. The asterisks indicate that the difference between ORF4^−^Tet^+^ and ORF4 revertant is statistically significant (p<0,05; Two-way ANOVA).

Infection of target cells by viruses is accompanied by virus-induced signaling events which create an appropriate intracellular environment to facilitate infection [35]. Thus, we set up experiments analyzing the phosphorylation status of a variety of serine/threonine kinases in infected cells. To this end, we simultaneously analyzed the relative phosphorylation of 14 kinases in NIH3T3 cells infected with parental virus or the mutant virus ORF4^−^Tet^+^ using the R&D Proteome Profiler® MAPK Array Kit. The results revealed phosphorylation of ERK1 and ERK2 after infection with either virus. In contrast, p38γ and particularly Akt (i.e., Akt1, Akt2 and Aktpan, but not Akt3) appeared strongly phosphorylated after infection with parental virus but only weakly after infection with ORF4^−^Tet^+^ (data not shown). Subsequently, we analyzed this in more detail applying cell-based protein phosphorylation ELISAs. While the initially observed difference in the phosphorylation of p38γ could not be verified in these subsequent experiments (data not shown), the difference in phosphorylation of Akt was consistently observed (Fig. 4A). Infection with both the ORF4^−^Tet^+^ mutant and the ORF4 revertant resulted in phosphorylation of Akt at 30 min after infection. However, while the level of phosphorylation continuously increased until 18 h after infection with the ORF4 revertant, the increase in the phosphorylation level was significantly delayed after infection with the ORF4^−^Tet^+^ mutant. Importantly, there was no difference in total Akt levels during the time course of the experiment (Fig. 4B). The decrease in total Akt levels (with both viruses) at later time points most likely reflects the reduction in overall cellular protein synthesis, indicative of host shutoff, as it has recently been published [36].

### Inhibition of Akt phosphorylation/activation by treatment with Triciribine

**Figure 5:**
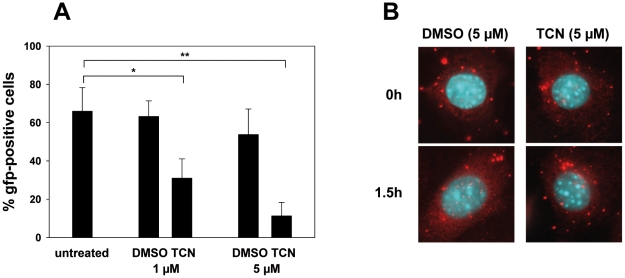
Inhibition of Akt activation in cells infected with parental virus leads to a phenotype closely resembling the phenotype of the ORF4 mutant virus. NIH3T3 cells were treated with the indicated doses of Triciribine (TCN), or as a control, with DMSO, starting 1 h prior to infection. Then, cells were infected at a multiplicity of infection of 5–10. After 1h at 37°C, the inoculum was removed and fresh medium, with TCN or DMSO, respectively, was added. Cells were harvested after 18 hours and analyzed for gfp expression by FACS analysis (panel A), or were analyzed after 1.5 hours by microscopy using a 100× oil immersion objective (panel B) as described in Figure 3. Data shown in panel A are means ± SD of four independent experiments. The asterisk indicates a statistically significant difference of p = 0,005, and the two asterisks indicate a statistically significant difference of p = 0,0003 (Student's t-test). In panel B, one representative experiment of 2 is shown.

These data suggested that activation of Akt is obviously important for the effective initiation of the infection. To support this hypothesis, we applied the well described specific inhibitor of Akt signaling Triciribine (TCN) [37]. Consistent with a role of Akt activation in the initial stage of infection, inhibition of Akt signaling by treatment of parental virus-infected cells with TCN resulted in a dose-dependent effect closely resembling the phenotype of the ORF4 mutant virus, as determined by FACS analysis and microscopy (Fig. 5A and B).

### Phosphorylation of Akt by insulin reverses the phenotype of the ORF4 mutant ORF4^−^Tet^+^

**Figure 6:**
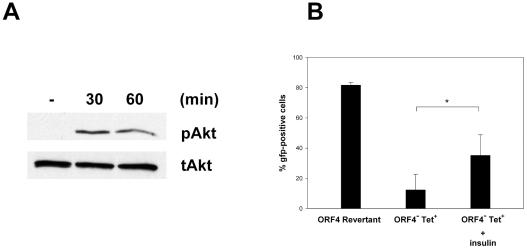
Phosphorylation of Akt by insulin reverses the phenotype of the MHV-68 ORF4 deletion mutant ORF4^−^Tet^+^. A) The expression of total Akt (tAkt) and its phosphorylated form (pAkt) was analyzed in insulin-treated NIH3T3 cells by Western blotting. The cells were treated with 10 µg/ml insulin for various time periods (min) as indicated. pAkt (upper panel) was detected with anti-phospho-Akt antibody (#9271), and tAkt (lower panel) was detected with anti-total-Akt antibody (#9272), both from Cell Signaling Technology (Danvers, MA). B) NIH3T3 cells were treated with insulin starting 60 min prior to infection. Then, insulin was washed away, and cells were infected at a multiplicity of infection of 5–10. After 1h at 37°C, the inoculum was removed and fresh medium was added. Cells were harvested after 18 hours and analyzed for gfp expression by FACS analysis. Treatment of the cells with insulin resulted in a significant reversion of the phenotype of the ORF4^−^Tet^+^ mutant (p = 0,037; Student's t-test). Data shown are means ± SD of at least three independent experiments.

We hypothesized that, if the initial signaling event is important, activation of Akt should reverse the phenotype of the ORF4 mutation. First, NIH3T3 cells were treated with insulin for 30 or 60 min, harvested and analyzed by Western blotting using anti-phospho-Akt antibody and anti-total-Akt antibody, respectively. As described [38], insulin readily induced phosphorylation of Akt (Fig. 6A). Second, we analyzed the effect of insulin treatment on viral infection. NIH3T3 cells were treated with insulin starting 60 min prior to infection. Then, insulin was washed away, and cells were infected at a multiplicity of infection of 5–10. After 1h at 37°C, the inoculum was removed and fresh medium was added. Cells were harvested after 18 hours and analyzed for gfp expression. As shown in Fig. 6B, treatment of the cells with insulin resulted in a significant, but not complete, reversion of the phenotype of the ORF4^−^Tet^+^ mutant.

### Complementation by ORF4 of KSHV

**Figure 7:**
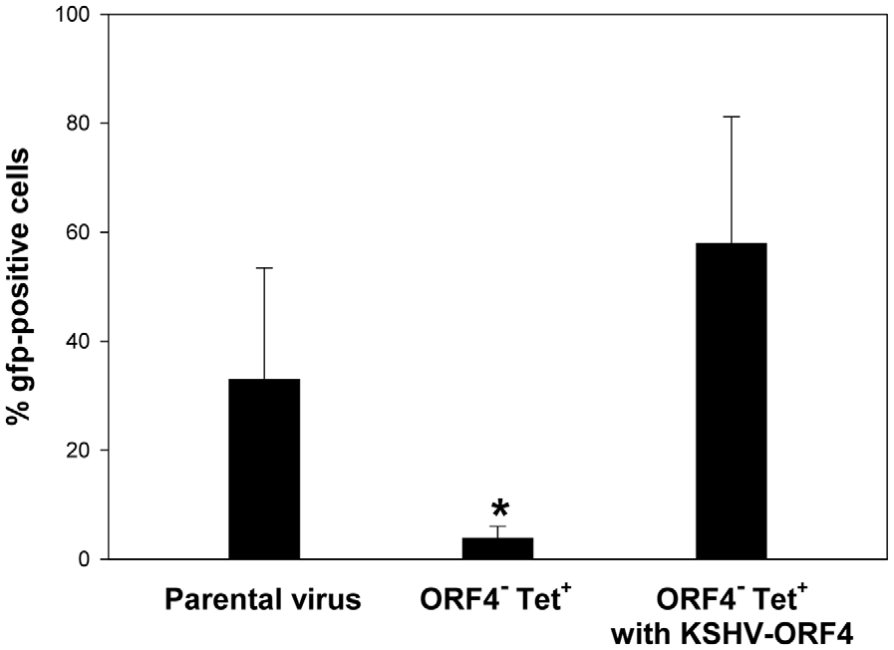
ORF4 of KSHV rescues the phenotype of the MHV-68 ORF4 deletion mutant ORF4^−^Tet^+^. Cells were infected at a multiplicity of infection of 10 for 1h at 37°C. Then, the inoculum was removed and fresh medium, containing phosphonoacetic acid (PAA), was added. Cells were harvested 18 hours after infection and analyzed for gfp expression by FACS analysis. Data shown are means ± SD of three independent experiments. The asterisk indicates that the number of gfp-positive cells after infection with the ORF4^−^Tet^+^ mutant was significantly reduced when compared to parental virus or ORF4^−^Tet^+^ with KSHV-ORF4 (p<0,05; Student's t-test).

To test whether ORF4 of KSHV can rescue the phenotype of an ORF4 deletion mutant, a recombinant MHV-68 expressing ORF4 of KSHV was constructed. For this purpose, ORF4 of KSHV was inserted in the mutant ORF4^−^Tet^+^. The insertion of ORF4 of KSHV did not change transcription levels of adjacent genes M1 and M2 (Fig. S2B). Cells were infected with parental virus, ORF4^−^Tet^+^ mutant virus and ORF4^−^Tet^+^ mutant virus expressing ORF4 of KSHV at a multiplicity of infection of 10 for 1h at 37°C. Then, the inocula were removed and fresh medium, containing phosphonoacetic acid (PAA), was added. Cells were harvested 18 hours after infection and analyzed for gfp expression by FACS analysis. As before, the number of gfp-positive cells after infection with the ORF4^−^Tet^+^ mutant was significantly reduced when compared to the infection with parental virus while expression of ORF4 of KSHV rescued the phenotype of the MHV-68 ORF4 deletion mutant ORF4^−^Tet^+^ (Fig. 7).

## Discussion

**Figure 8:**
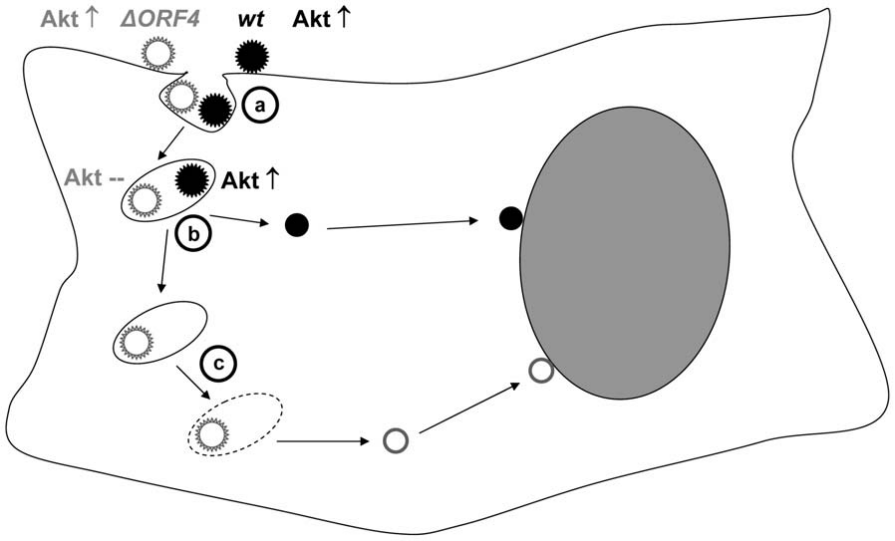
Current model/Working hypothesis. Both wildtype virus (wt; black) and ORF4 mutant virus (ΔORF4; grey) initially activate – in an ORF4-independent manner – the Akt pathway (a). After being endocytosed, the wt virus is able to maintain the activation of Akt in an ORF4-dependent manner which aids to leave the endosome rather quickly (b). In contrast, due to the lack of ORF4, ORF4 mutant virus is trapped much longer in the endosome and escapes with delay, most likely by additional, ORF4-independent mechanisms (c).

Previously, it had been shown that the MHV-68 RCA protein inhibits complement activation at the level of C3 [18]. It plays an important role in viral evasion from complement in acute, persistent and latent infection in vivo [23]. Furthermore, it was shown to be the major glycosaminoglycan (GAG)-binding protein of MHV-68 [29], promoting the initial contact of virions to cellular GAGs [39]. Recently, it has also been shown to facilitate replication in macrophages in a complement independent manner, being required for efficient DNA synthesis [30]. Here, we elucidated a new function of the MHV-68 RCA protein, encoded by ORF4. While, as reported by others [40], attachment and penetration of virions lacking the RCA protein were not affected, the entry of the virus genome into the nucleus of the infected cell was slowed down by mutation of ORF4. This finding explains our previously reported observation of delayed growth of the ORF4^−^Tet^+^ mutant in fibroblasts in cell culture [32]. In contrast to our findings, another group did not observe the delayed growth of ORF4-deficient virus (ORF4.STOP) on fibroblasts [23], [30]. We assume that this might be due to a different basal Akt activation status of the cells used and/or to the different infection protocols. As described [32], we infected the cells for 1h at 4°C to allow adsorption. For penetration, prewarmed medium was then added for a 2h incubation period at 37°C. Finally, remaining extracellular virus was inactivated by low pH-treatment with citrate buffer for 1min. Thus, the infection is highly synchronized and remaining input virus is more vigorously removed than after standard infection procedures which may allow to uncover differences at very early steps of infection. In our assays (Fig. 2 and 3), the ORF4.STOP mutant showed a phenotype very similar to the phenotype of the ORF4^−^Tet^+^ mutant. Analysis of the phosphorylation status of a variety of kinases in infected cells revealed a significant reduction in the phosphorylation of the protein kinase Akt in cells infected with ORF4 mutant virus. Consistent with a role of Akt activation in the initial stages of infection, inhibition of Akt signaling resulted in a similar phenotype as observed for the ORF4 mutant virus. Vice versa, activation of Akt by insulin reversed the phenotype of the ORF4 mutant to a significant extent. Interestingly, it had been speculated earlier that the MHV-68 RCA protein might function through induction of intracellular signals [18]. The homologous ORF4 of KSHV was able to rescue the phenotype of the MHV-68 ORF4 mutant, indicating that ORF4 is functionally conserved and that ORF4 of KSHV might have a similar function in infection initiation. The RCA proteins of MHV-68 and of KSHV are virion constituents [28], [29]. Thus, it is conceivable that they can trigger signal transduction cascades which might facilitate productive infection. Consistent with our data, it has recently been shown that MHV-68 infection of NIH3T3 cells activates Akt [41]. Although it was postulated that early Akt activation possibly correlated with viral binding and entry, the authors did not observe a significant effect of the PI3K inhibitor LY294002 on early steps of the viral life cycle. In our hands, treatment of cells with the Akt inhibitor TCN resulted in a delayed delivery of the viral genome to the nucleus of infected cells. In addition, ORF4 mutant viruses with a reduced capacity to activate Akt showed the same phenotype, and activation of Akt by insulin was able to release this phenotype to a significant extent, thus providing further evidence for a role of Akt activation in promoting infection. For KSHV, it has been shown that the appropriate intracellular environment to facilitate infection is created by the induction of various signaling cascades: KSHV can induce the PI3-kinase-PKC-ξ-MEK-ERK signaling pathway early during infection [42], [43]. It also induces the integrin-dependent focal adhesion kinase-Src-PI3-kinase-Rho-GTPase signaling pathway by its glycoprotein gB [44]–[46]. In addition, KSHV also activates the JNK and p38 mitogen-activated protein kinase (MAPK) pathways during primary infection [47]. Many of these signaling events have been shown to be important for post-binding virus entry steps, for example modulation of microtubules, movement of virus in the cytoplasm and nuclear delivery of viral DNA [48]. The K1 protein of KSHV activates the Akt signaling pathway in B lymphocytes and it was suggested that it thereby promotes cell survival and prevents apoptosis of infected cells [49]. Many viruses and viral proteins affect the PI3-kinase-Akt signaling pathway, and this has mainly been associated with its fundamental role in the regulation of apoptosis and cellular survival [50]. However, besides its central role in regulation of apoptosis, the PI3-kinase-Akt signaling pathway is also involved in the regulation of numerous cellular functions as diverse as cell metabolism, cell polarity and membrane and vesicular trafficking [51], [52]. Thus, activation of this pathway may be exploited by viruses in multiple ways beyond the regulation of apoptosis. Indeed, human cytomegalovirus up-regulates the PI3-kinase pathway to initiate viral DNA replication and to complete the lytic life cycle [53]. Importantly, similar to our data, it has recently been shown that the PI3-kinase-Akt pathway controls cellular entry of Ebola virus [54]. Inhibition of PI3-kinase or Akt caused an aberrant accumulation of Ebola virus particles in intracellular vesicles, indicating a role of the PI3-kinase-Akt pathway in vesicular trafficking of virus particles [54]. Entry of KSHV and MHV-68 into fibroblasts or epithelial cells occurs via endocytosis [55]–[57]. Endocytosed viruses then penetrate the endosomal membrane by fusion of the viral envelope with the membrane of the endocytic vesicle to be released into the cytoplasm [35]. Thus, we hypothesize that virus lacking the RCA protein is less efficient in its ability to leave endocytic vesicles. Our current working hypothesis (Fig. 8) is that both wt and ORF4 mutant virus initially activate – in an ORF4-independent manner - the Akt pathway. After being endocytosed, the wt virus is able to maintain the activation of Akt in an ORF4-dependent manner which aids to control endocytic movement and/or to leave the endosome rather quickly. In contrast, the ORF4 mutant virus is trapped much longer in the endosome and escapes with delay, most likely by additional, ORF4-independent mechanisms. Since the RCA protein encoded by ORF4 is incorporated into mature viral particles via interactions with other virion components, it might also be possible that mutation of ORF4 additionally affects other viral proteins which could also contribute to the observed effects. It is well known that viruses may alter cellular signaling pathways, in particular PI3-kinase, to control their endocytic movement [35]. For example, endocytosis of adeno-associated virus type 2 occurs through activation of Rac1 and PI3-kinase which directs virus movement along the cytoskeleton to the nucleus [58]. In addition, inhibition of adenovirus type 2-mediated signaling via protein kinase C has been shown to prevent escape of adenovirus from endosomes, resulting in an accumulation in cytoplasmic vesicles near the cell periphery [59]. It is also known that endocytic vesicles can function as a signaling compartment distinct from the plasma membrane [60]. Signaling from endosomes allows spatial and temporal regulation of signal transduction, and the transmitted signals may be qualitatively different from those initiated at the plasma membrane [60], [61]. We hypothesize that this is the reason why activation of Akt by insulin did not completely reverse the phenotype of the ORF4 mutant. Addition of insulin to the cell culture can certainly not completely mimic signaling from endosomes. The KSHV-K1 protein continues to transduce Akt-activating signals from an intracellular compartment after internalization through clathrin-mediated endocytosis [62], providing a paradigm for a gammaherpesviral protein which signals from endocytic vesicles. Since Akt is known to be associated with certain vesicles [63], it is plausible that viral proteins may activate Akt from intracellular compartments.

In summary, our studies demonstrate the ORF4 contributes to an efficient de novo infection process and thus reveal a novel function of a gammaherpesvirus RCA protein. This novel function might also contribute, besides the already known functions described above, to the lytic replication deficit which has been observed in vivo after intracerebral and intraperitoneal [23] or intranasal [40] infection of mice with ORF4 mutant viruses. Notably, in a meningoencephalitis model of acute MHV-68 infection of weanling mice, the MHV-68 RCA protein was observed to play a key role in the invasion of brain parenchymal cells [23]. It was also observed that the MHV-68 RCA protein was particularly present in the cytoplasm and plasmalemma of infected CNS cells, however, the potential physiologic role of cytoplasmic MHV-68 RCA protein remained unclear. It is tempting to speculate that in this model, the Akt-activating function described here may have contributed to the observed phenotype. It may thus provide a rationale for the observation of Kapadia et al. that the most significant attenuation caused by the loss of the MHV-68 RCA protein was observed in the CNS, an organ where complement levels are actually considered to be rather low [23].

## Materials and Methods

### Cell culture, virus stocks and plaque assays

Cell culture, virus stocks and plaque assays were performed essentially as described [32].

### Generation of recombinant viruses

The construction of the mutants ORF4^−^Tet^+^ and ORF4^−^Tet^−^ and of the corresponding revertant (ORF4 revertant) has already been described [32]. To test whether ORF4 of KSHV can rescue the phenotype of the mutant ORF4^−^Tet^+^, ORF4 of KSHV was inserted in the mutant ORF4^−^Tet^+^. For this purpose, ORF4 of KSHV (kindly provided by Dr. J. Haas, Max von Pettenkofer-Institute, Munich, Germany) was cloned into the plasmid pMCMV4 (kindly provided by Dr. M. Messerle, Hannover Medical School, Hannover, Germany) between the MCMV promoter and polyA signal. The expression cassette (excised by HindIII) was cloned blunt end into the BglII site (position 3846 of the MHV-68 genome) of the plasmid pST76K-SR containing a 3.9 kb fragment of MHV-68 (nucleotide positions 2406–6261). The resulting shuttle plasmid was used for mutagenesis by the two-step replacement procedure as described [64], [65]. For comparison, we additionally tested an independently constructed ORF4 mutant (ORF4.STOP) [23], kindly provided by Dr. H.W. Virgin (Washington University, MO), containing a translational stop in ORF4 which eliminates expression of the MHV-68 RCA protein.

### FACS Analysis

NIH3T3 cells (ATCC CRL 1658) were infected at a multiplicity of infection of 10 for 1h at 37°C. Then, the inoculum was removed and fresh medium, with or without phosphonoacetic acid (PAA), was added. PAA, an inhibitor of DNA synthesis, was used at a final concentration of 250 µg/ml. Cells were harvested immediately thereafter and after 6, 18 and 48 hours, respectively, centrifuged and resuspended in 0.5% to 2% paraformaldehyde in PBS. Usually, 5000–10000 cells were collected on a FACSCalibur (Becton Dickinson, Heidelberg, Germany) and analyzed for gfp expression. Data analysis was performed using CellQuest™ software (Becton Dickinson).

### RNA-isolation and RT-PCR

Total RNA was isolated from infected cells using the RNeasy® Mini Kit (Qiagen GmbH, Hilden, Germany), followed by an additional DNAse treatment using the TURBO DNA-free™ Kit (Applied Biosystems, Darmstadt, Germany), according to the instructions of the manufacturers. 500 ng RNA was reverse transcribed using the High Capacity cDNA Reverse Transcription Kit (Applied Biosystems) according to the instructions of the manufacturer or subjected to mock reverse transcription in the absence of the enzyme (-RT control). 5 µl of the resulting cDNA were used as template for PCR amplification with the ABI 7300 Real Time PCR System, using Power SYBR® Green PCR Master Mix and universal cycling conditions (Applied Biosystems). C_t_ values of -RT controls did not exceed background levels. The following MHV-68 gene specific primers were used:

ORF50 forward: 5′-CCCACGGTTCGCTATACAGTAAAGAC-3′

ORF50 reverse: 5′-ATTGTGTAGAGGGTCCAGGTTAATGATTTC-3′

M1 forward: 5′-ATCTCACCTTTGCTGGATTCTTATTTGC-3′

M1 reverse: 5′-GTTCTGATGGCTTGAAACGATGGC-3′

M2 forward: 5′-TCCTCGCCCCACTCCACAAAAC-3′

M2 reverse: 5′-AACACCCCATGAACCCTGAGATACG-3′

M4 forward: 5′-CCTCGGCATGGGATAACTATACTTCTG-3′

M4 reverse: 5′-GCTGTGTGGCATTTGAACCTCTTG-3′

ORF6 forward: 5′-TTCAATGTCAAGGCACCGTGTCAG-3′

ORF6 reverse: 5′-CTTGTTACTGTTGTGAAAGACGATGGC-3′.

As a housekeeping gene, the murine ribosomal protein L8 gene (rpl8) was amplified in parallel using the following primers:

L8 forward: 5′-CAGTGAATATCGGCAATGTTTTG-3′

L8 reverse: 5′-TTCACTCGAGTCTTCTTGGTCTC-3′.

### Assays for virus attachment and penetration

Attachment of viruses to NIH3T3 cells was assessed by two different methods: i) Virus suspensions were incubated with NIH3T3 cells for one hour at 37°C. Then, the cells were pelleted by centrifugation, and the supernatant was collected. Afterwards, the amount of virus in the original suspension (before incubation with cells) as well as in the supernatant (after incubation with cells) was determined by plaque assay. The reduction of the virus titer was taken as the amount of attached virus. ii) Direct binding assays using radiolabeled virus were performed as described by others [66]–[68]. Viruses were labeled by generating virus stocks in the presence of ^3^H-thymidine. Cells were incubated with comparable amounts of radiolabeled viruses for 2 hours at 4°C, extensively washed three times with ice-cold PBS at 4°C to remove unbound viruses, harvested and lysed in 1% SDS. The cell-associated radioactivity of the lysates was determined by counting in a scintillation counter. The cell-associated radioactivity in percent of the input (as determined by counting the radioactivity of the input virus) was taken as the amount of attached virus.

For the determination of the penetration kinetics of the various viruses, two different methods which have been described by others [67], [69] were applied: i) NIH3T3 cells were incubated with virus for one hour at 4°C to allow adsorption. The inoculum was then removed, and prewarmed medium was added at 37°C to allow penetration. Immediately thereafter and after 5, 10, 30, 60, 240 and 480 min, remaining extracellular virus was inactivated by low-pH treatment with citrate buffer for 1min. Cells were washed with PBS and fresh medium containing 1.5% carboxymethylcellulose was added. Cells were stained after 4 to 5 days with 0.1% crystal violet solution to determine the number of plaques. Plaques were counted, and the percentage of PFU surviving low-pH treatment, compared to a PBS-treated control, was calculated. ii) NIH3T3 cells were incubated with radiolabeled virus as described above for one hour at 4°C to allow adsorption. The inoculum was then removed, and prewarmed medium was added at 37°C to allow penetration. Immediately thereafter and after 1, 4 and 8 hours, remaining extracellular virus was removed by extensive treatment with trypsin. Cells were washed twice with PBS and the radioactivity of the lysates, reflecting the fraction of internalized virus, was determined by counting in a scintillation counter. The percentage of trypsin-resistant, i.e. internalized virus, compared to a PBS-treated control, was calculated.

### Assays for the determination of kinase phosphorylation

For an initial screen of the phosphorylation status of a variety of serine/threonine kinases in infected cells, we used the R&D Proteome Profiler® MAPK Array Kit (R&D Systems, Wiesbaden, Germany). This Kit allows the simultaneous analysis of the relative phosphorylation of 14 kinases in murine cells (ERK1 and 2, JNK1 and 2, MSK2, RSK2, p38α, γ and δ, GSK-3α/β and GSK-3β, Akt1, 2 and 3) and was used according to the instructions of the manufacturer. After the initial screening, selected kinases were analyzed in more detail using cell-based protein phosphorylation ELISA Kits (Cellular Activation of Signaling ELISA [CASE™ Kit]) (SuperArray Bioscience Corporation, Frederick, MD) according to the instructions of the manufacturer.

### Inhibition of Akt signaling

For the inhibition of Akt signaling, we used the well described inhibitor Triciribine (TCN) which inhibits Akt signaling in a very selective fashion [37]. TCN (Biaffin GmbH, Kassel, Germany) was dissolved in DMSO to prepare a 3 mM stocksolution and was used in final concentrations of 1 and 5 µM. NIH3T3 cells were treated with TCN, or as a control, with DMSO, starting 1 h prior to infection. Then, cells were infected at a multiplicity of infection of 5–10. After 1h at 37°C, the inoculum was removed and fresh medium, with TCN or DMSO, respectively, was added. Cells were harvested after 18 hours, centrifuged, resuspended in 0.5% to 2% paraformaldehyde in PBS and analyzed for gfp expression as described above.

### Activation of Akt signaling by treatment with insulin

To activate Akt signaling, we used insulin (SIGMA, Taufkirchen, Germany) at a final concentration of 10 µg/ml which had been shown to activate Akt in NIH3T3 cells [38]. First, to demonstrate phosphorylation of Akt in NIH3T3 cells by insulin, cells were treated for 30 or 60 min, harvested and analyzed by Western blotting using anti-phospho-Akt antibody (#9271) and anti-total-Akt antibody (#9272), respectively (Cell Signaling Technology, Inc., Danvers, MA). Second, to analyze the effect of insulin treatment on viral infection, NIH3T3 cells were treated with insulin starting 60 min prior to infection. Then, insulin was washed away, and cells were infected at a multiplicity of infection of 5–10. After 1h at 37°C, the inoculum was removed and fresh medium was added. Cells were harvested after 18 hours, centrifuged, resuspended in 0.5% to 2% paraformaldehyde in PBS and analyzed for gfp expression as described above.

### Immunofluorescence staining of infected cells

For immunofluorescence staining, NIH3T3 cells were seeded on coverslips in 12 well plates the day before staining. Cells were infected at a multiplicity of infection of 5–10 for 1h at 4°C to allow adsorption. Then, cells were washed three times, prewarmed medium was added and the cells were incubated at 37°C. At the indicated time points, cells were fixed with 4% paraformaldehyde and permeabilized with 0.1% Triton-X100. Virus particles were visualized with polyclonal rabbit anti-MHV-68 antiserum followed by goat anti-rabbitCy3 antibody (Chemicon, Hofheim, Germany). Stained cells were mounted in ProLong® Gold antifade reagent with DAPI (Molecular Probes Inc., Eugene, OR) and analysed by using a Zeiss Axiovert 200M microscope.

## Supporting Information

Table S1 Deletion of ORF4 does not influence attachment.
(0.05 MB DOC)

Figure S1 The phenotype of ORF4 deletion mutants is not cell type specific. NIH3T3 or C57BL/6 mouse embryonic fibroblasts (MEF) (ATCC SCRC-1008) were infected at a multiplicity of infection of 10 for 1h at 37°C. Then, the inoculum was removed and fresh medium was added. Cells were harvested 18 hours after infection and analyzed for gfp expression by FACS analysis. For better comparison, the number of gfp-positive cells after infection with the revertant virus was set to 100%. Data shown are means +/− SD of three independent experiments. The asterisks indicate that the number of gfp-positive cells after infection with the ORF4 deletion mutants was significantly reduced when compared to revertant virus (p<0,0005; Student's t-test).
(0.12 MB TIF)

Figure S2 Quantitative RT-PCR analysis of M4 and ORF 6 (panel A) and M1 and M2 (panel B) transcription in NIH3T3 cells infected with the indicated viruses. Shown are means +/− SD of three independent experiments. L8 transcription was analyzed in parallel as a control.
(0.17 MB TIF)

Figure S3 Determination of penetration kinetics. NIH3T3 cells were incubated with virus for one hour at 4°C to allow adsorption. The inoculum was then removed, and prewarmed medium was added at 37°C to allow penetration. Immediately thereafter and after 5, 10, 30, 60, 240 and 480 min, remaining extracellular virus was inactivated by low-pH treatment with citrate buffer for 1min. Cells were washed with PBS and fresh medium containing 1.5% carboxymethylcellulose was added. Cells were stained after 4 to 5 days with 0.1% crystal violet solution to determine the number of plaques. Plaques were counted, and the percentage of PFU surviving low-pH treatment, compared to a PBS-treated control, was calculated. Data shown are means +/− sem from two independent experiments. No significant differences were observed between parental virus, ORF4-Tet+ and ORF4 revertant.
(0.11 MB TIF)
