# Supplementary material for: A Gammaherpesvirus Complement Regulatory Protein Promotes Initiation of Infection by Activation of Protein Kinase Akt/PKB
Source: PLoS One. 2010 Jul 21;5(7):e11672. doi: 10.1371/journal.pone.0011672 (PMC2908122; doi:10.1371/journal.pone.0011672)
Supplement: Table S1 — Deletion of ORF4 does not influence attachment. (0.05 MB DOC) [file pone.0011672.s001.doc]

**Table S1:** Deletion of ORF4 does not influence attachment.

|  | **Input ^(*)^** | **Cell-associated ^(*)^** | **Attached**  **(% of input)** |
| --- | --- | --- | --- |
| **Experiment 1** |  |  |  |
|  |  |  |  |
| **Parental virus** | 245620 + 73337 | 23754 + 6996 | 9.7 + 2.8 |
| **ORF4^-^Tet^+^** | 195268 + 42745 | 18292 + 1228 | 9.4 + 0.6 |
| **ORF4 revertant** | 208028 + 62295 | 15853 + 613 | 7.6 + 0.3 |
|  |  |  |  |
| **Experiment 2** |  |  |  |
|  |  |  |  |
| **Parental virus** | 110647 + 13694 | 13153 + 3572 | 11.9 + 3.2 |
| **ORF4^-^Tet^+^** | 80974 + 13782 | 10387 + 1262 | 12.8 + 1.6 |
| **ORF4 revertant** | 95313 + 13690 | 9071 + 1706 | 9.5 + 1.8 |

Direct binding assays using radiolabeled virus were performed as described in materials and methods. Viruses were labeled by generating virus stocks in the presence of ^3^H-thymidine. The cell-associated radioactivity in percent of the input was taken as the amount of attached virus. Two independent experiments are depicted. Data shown are means + SD from triplicates. No significant differences were observed between parental virus, ORF4^-^Tet^+^ and ORF4 revertant. ^(*)^ ^3^H-thymidine counts
