# Supplementary figures and images for: A Gammaherpesvirus Complement Regulatory Protein Promotes Initiation of Infection by Activation of Protein Kinase Akt/PKB
Source: PLoS One. 2010 Jul 21;5(7):e11672. doi: 10.1371/journal.pone.0011672 (PMC2908122; doi:10.1371/journal.pone.0011672)

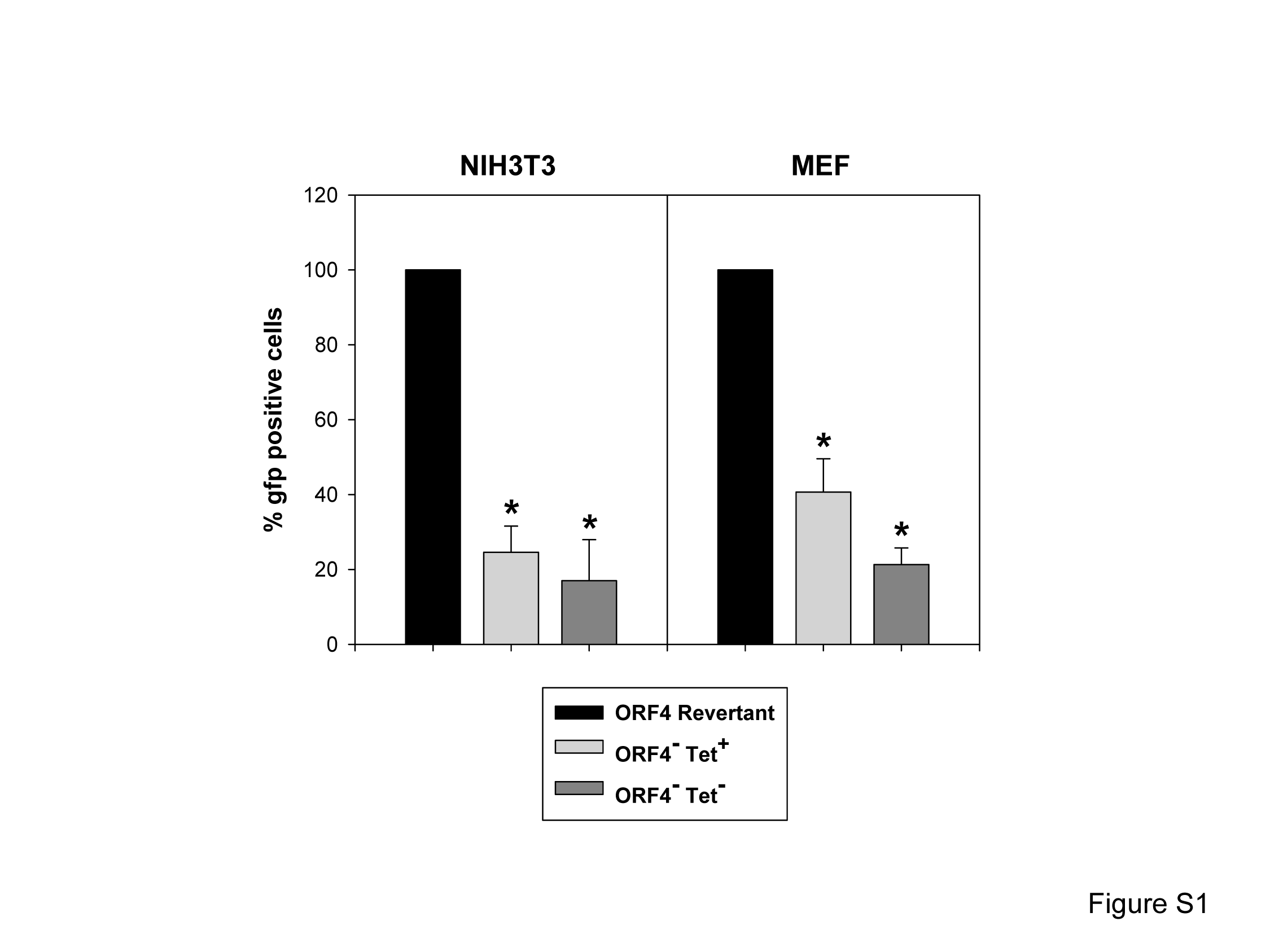

Supplement: Figure S1 — The phenotype of ORF4 deletion mutants is not cell type specific. NIH3T3 or C57BL/6 mouse embryonic fibroblasts (MEF) (ATCC SCRC-1008) were infected at a multiplicity of infection of 10 for 1h at 37°C. Then, the inoculum was removed and fresh medium was added. Cells were harvested 18 hours after infection and analyzed for gfp expression by FACS analysis. For better comparison, the number of gfp-positive cells after infection with the revertant virus was set to 100%. Data shown are means +/− SD of three independent experiments. The asterisks indicate that the number of gfp-positive cells after infection with the ORF4 deletion mutants was significantly reduced when compared to revertant virus (p<0,0005; Student's t-test). (0.12 MB TIF) [file pone.0011672.s002.tif]

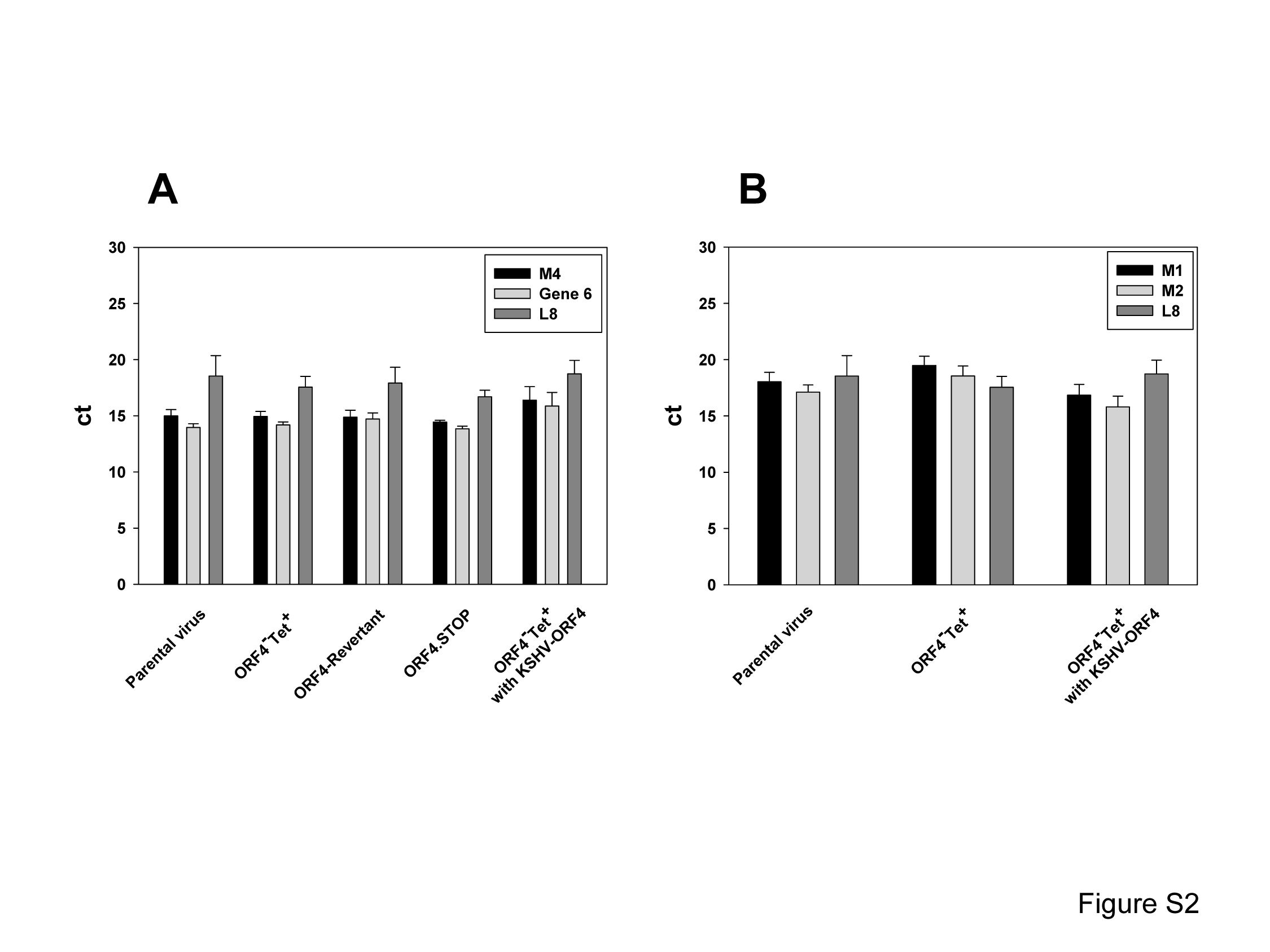

Supplement: Figure S2 — Quantitative RT-PCR analysis of M4 and ORF 6 (panel A) and M1 and M2 (panel B) transcription in NIH3T3 cells infected with the indicated viruses. Shown are means +/− SD of three independent experiments. L8 transcription was analyzed in parallel as a control. (0.17 MB TIF) [file pone.0011672.s003.tif]

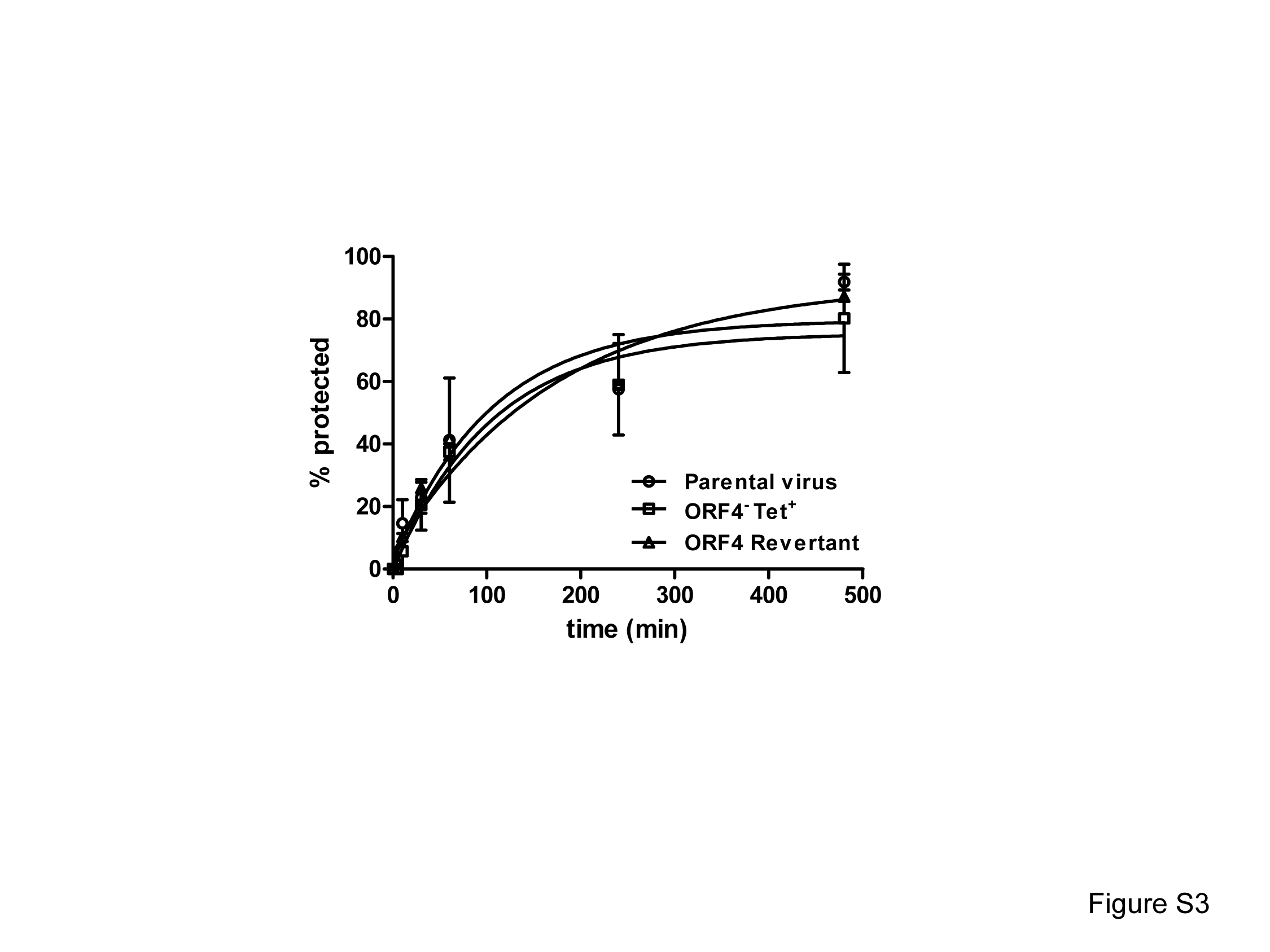

Supplement: Figure S3 — Determination of penetration kinetics. NIH3T3 cells were incubated with virus for one hour at 4°C to allow adsorption. The inoculum was then removed, and prewarmed medium was added at 37°C to allow penetration. Immediately thereafter and after 5, 10, 30, 60, 240 and 480 min, remaining extracellular virus was inactivated by low-pH treatment with citrate buffer for 1min. Cells were washed with PBS and fresh medium containing 1.5% carboxymethylcellulose was added. Cells were stained after 4 to 5 days with 0.1% crystal violet solution to determine the number of plaques. Plaques were counted, and the percentage of PFU surviving low-pH treatment, compared to a PBS-treated control, was calculated. Data shown are means +/− sem from two independent experiments. No significant differences were observed between parental virus, ORF4-Tet+ and ORF4 revertant. (0.11 MB TIF) [file pone.0011672.s004.tif]
